# Mobile phone use for pregnancy-related healthcare utilization and its association with optimum antenatal care and hospital delivery in Bangladesh

**DOI:** 10.1371/journal.pgph.0001762

**Published:** 2023-04-06

**Authors:** Gulam Muhammed Al Kibria, Mohammad Rashidul Hashan, Abu Abdullah Mohammod Hanif, Vidhi Maniar, Md Shajedur Rahman Shawon

**Affiliations:** 1 Johns Hopkins Bloomberg School of Public Health, Baltimore, MD, United States of America; 2 Bangladesh Civil Service, Ministry of Health and Family Welfare, Government of Bangladesh, Dhaka, Bangladesh; 3 Central Queensland University, Rockhampton, Australia; 4 BRAC James P Grant School of Public Health, BRAC University, Dhaka, Bangladesh; 5 Centre for Big Data Research on Health, University of New South Wales, Sydney, Australia; University of Washington, UNITED STATES

## Abstract

Pregnancy-related healthcare utilization is inadequate in Bangladesh, where more than half of pregnant women do not receive optimum number of antenatal care (ANC) visits or do not deliver child in hospitals. Mobile phone use could improve such healthcare utilization; however, limited evidence exists in Bangladesh. We investigated the pattern, trends, and factors associated with mobile phone use for pregnancy-related healthcare and how this can impact at least 4 ANC visits and hospital delivery in the country. We analyzed cross-sectional data from Bangladesh Demographic and Health Survey (BDHS) 2014 (n = 4,465) and 2017–18 (n = 4,903). Only 28.5% and 26.6% women reported using mobile phones for pregnancy-related causes in 2014 and 2017–18, respectively. Majority of the time, women used mobile phones to seek information or to contact service providers. In both survey periods, women with a higher education level, more educated husbands, a higher household wealth index, and residence in certain administrative divisions had greater likelihoods of using mobile phones for pregnancy-related causes. In BDHS 2014, proportions of at least 4 ANC and hospital delivery were, respectively, 43.3% and 57.0% among users, and 26.4% and 31.2% among non-users. In adjusted analysis, the odds of utilizing at least 4 ANC were 1.6 (95% confidence interval (CI): 1.4–1.9) in BDHS 2014 and 1.4 (95% CI: 1.3–1.7) in BDHS 2017–18 among users. Similarly, in BDHS 2017–18, proportions of at least 4 ANC and hospital delivery were, respectively, 59.1% and 63.8% among users, and 42.8% and 45.1% among non-users. The adjusted odds of hospital delivery were also high, 2.0 (95% CI: 1.7–2.4) in BDHS 2014 and 1.5 (95% CI: 1.3–1.8) in BDHS 2017–18. Women with history of using mobile phones for pregnancy-related causes were more likely to utilize at least 4 ANC visits and deliver in health facilities, however, most women were not using mobile phones for that.

## Introduction

Globally, maternal and neonatal deaths declined substantially during the past couple of decades, however, the maternal mortality ratio (MMR) and neonatal mortality rate (NMR) remain high in many low- and middle-income countries (LMICs) [1, 2]. Most maternal and neonatal deaths also occur in LMICs [1–3]. Moreover, a major proportion of women in LMICs neither receive optimum number of antenatal care (ANC) visits during pregnancy nor utilize health facilities for delivery. Appropriate and timely provision of such health services are important to prevent maternal and neonatal deaths [4, 5]. Bangladesh, an LMIC of South Asia, is also experiencing a substantially higher MMR (173 per 100,000 live-births in 2017) and NMR (30 deaths per 1000 live-births in 2017–18) in recent years [2, 6]. Additionally, Bangladesh Demographic and Health Survey (BDHS) 2017–18 showed that less than half of the women received at least four ANC visits (47%) or underwent hospital delivery (49%) [6]. The sustainable development goals (SDGs) aim to reduce the MMR and NMR to 70 per 100,000 live-births and 12 per 1,000 live-birth within 2030, respectively [7]. It is crucially important for Bangladesh to scale up the ANC visits and/or increase hospital delivery to achieve the SDG defined targets within this timeline.

Globally, the ubiquitous presence of mobile phones, has been a phenomenal tool providing healthcare services delivery and information in low-resource settings [8, 9]. Several newer terms such as mobile health (mHealth), electronic health (eHealth), telehealth, and telemedicine are in use to describe usages of mobile and desktop technology for health care purposes [10, 11]. According to the World Bank, the number of mobile subscribers was 0.22 per 100 people during the year 2000 among Bangladeshi people which exponentially increased to 46, 84, and 103 in 2010, 2015, and 2020, respectively [12]. Similarly, according to the BDHS 2014, a nationally representative survey conducted in the country, approximately 52% of males and 18% of females owned a mobile phone [13]. According to the 2017–18 BDHS, those proportions were 74% and 47% for males and females, respectively [6]. Though mobile phones are currently not utilized by health facilities to schedule ANC visits or child delivery purposes, successful utilization of such reminders for the coronavirus disease 2019 (COVID-19) pandemic vaccine scheduling has made it possible to use that for other health care services (e.g., maternal health care) in this country [14]. A growing body of literature demonstrates the importance of mobile phone use for improving health outcomes in Bangladesh and other LMICs. Tang et al. analyzed BDHS 2014 data and found that women who used their mobile phones for pregnancy-related purposes were more than twice as likely to receive at least three ANC visits or give birth in a hospital [15]. Using mobile phones can also increase access to healthcare in hard-to-reach areas. It can also serve as an educational tool for mothers. However, only a quarter of the women used it for pregnancy-related causes [13, 15]. Identifying the factors associated with mobile phone use for pregnancy-related causes can be used to design appropriate interventions to increase its use among women with lower usage. Due to an increase in mobile phone subscription and ownership following that survey, it is important to examine the trend in mobile phone use for pregnancy-related causes and its association with maternal health care services utilization in the country. Although some research has been carried out on these topics, there remains a lack of updated data in many countries, including Bangladesh [15, 16]. This limits our understanding of mobile phone use to design evidence-based programs and shows that its use has been underestimated here. Using two nationally representative surveys from Bangladesh, BDHS 2014 and 2017–18, we attempted to fill these knowledge gaps in this country.

This study has three primary objectives: (1) to describe the mobile phone usage prevalence for pregnancy-related causes; (2) to identify the sociodemographic factors associated with this use; and (3) to investigate the association of mobile phone for pregnancy-related causes with at least 4 ANC visits and hospital delivery. Findings of the present study will be useful for researchers and policymakers to expand the mobile phone use for maternal health care utilization in Bangladesh.

## Methods

### Ethics statement

BDHS 2014 and 2017–18 received ethical approval from the International Institute for Population Science and ICF International Institutional Review Boards. All adult respondents (i.e., at least 18-year-olds) provided informed consent to participate. For participants with 15–17 years of age, consent was obtained from a legal guardian; the respondents provided assent as well. In December 2020, electronic approval was obtained to use data from ICF International, Rockville, Maryland, USA.

### Study design

We analyzed BDHS 2014 and BDHS 2017–18 data. These cross-sectional surveys primarily aim to collect data on major demographic and health indicators in Bangladesh, including indicators of maternal and child health. BDHS covers rural and urban regions in all divisions (i.e., the largest administrative units). Both surveys were conducted by Mitra and Associates, a private research organization in Bangladesh. About two hundred data collectors were trained and recruited. BDHS 2014 data were collected from June to November 2014 while data collection for BDHS 2017–18 took place from October 2017 to March 2018. Ever-married women of reproductive-age (i.e., 15-49-year-olds) were interviewed with the women’s questionnaire [6, 13].

### Sample design and coverage

First, a sampling frame was preapred with a list of enumeration regions (EAs). This list was prepared based on housing and population census 2011 in the country. Women were interviewed in households and this list of households were selected in two stages. During BDHS 2014, 393 EAs were selected from rural regions and 207 EAs were selected from urban regions. For BDHS 2017–18, a total of 425 and 250 EAs were selected from rural and urban regions, respectively. Next, a sample of 30 houesholds were selected from each EA. In this way, a naionally representative sample were otained to reflect statistically reliable estimates of the surveyed indicators. The survey results, methodology, questionnaire, and sample size estimates are reported elsewhere [6, 13]. BDHS 2014 interviewed 18,245 reproductive-age women from 17,989 selected households [13]. During BDHS 2017–18, a total of 20,127 reproductive-age women were interviewed from 20,160 slected households [6]. The response rate was 98% [6, 13]. We studied the last deliveries occurring in the past three years prior to the survey to minimize recall bias. S1 Fig describes the sample extraction process for both years.

### Study variables

Women were asked if they or any of their family members used mobile phones to obtain health services or advice during pregnancy or delivery. Then, they were asked the reason for using: “ask what to do”; “contact service provider”; “arrange transport”; “arrange money”; “arrange the place of delivery”; and “others” [6, 13]. Throughout this paper, the term ’user group’ refers to women who received any services via mobile phones for pregnancy-related reasons, whereas the term ’non-user group’ refers to women who did not receive any services via mobile phones.

Women were questioned about whether they received any ANC during their pregnancy (i.e., "Did you see anyone for antenatal care during this pregnancy?"). They were also asked the number of times they received ANC visits. We dichotomized this based on receiving at least 4 ANC visits (i.e., yes or no). The location of delivery (i.e., at home or in a hospital) was determined by asking respondents about their most recent delivery (i.e., "Where did you give birth to… .?"). Hospital deliveries were defined as those that occurred in a hospital. Home deliveries were defined as those made to a woman’s residence, a friend’s residence, or a relative’s residence [6, 13].

Following potential associated factors and confounders were selected based on literature review, scientific plausibility, and data structure: women’s age, parity, women’s education, husband’s education, current work status, religion, household wealth status, and rural-urban place and division of residence. S2 Fig describes the conceptual framework we used for the present study. Maternal age was grouped into <20, 20–34, and 35–49 years. Women reported the number of times they have become pregnant in their life, it was categorized as ‘primi’ (i.e., first pregnancy) and ‘2 or more’. Women reported their education level, husband’s education level, work status, place (i.e., rural or urban), and division of residence. Education level was grouped as follows: no formal education, primary (i.e., 1 to 5 school years), secondary (i.e., 6 to 10 school years), and college or above (i.e., 11 or more school years). Household wealth index score was obtained using principal component analysis of basic household construction materials, sources of water, sanitation facilities, electricity, and household belongings; then, the score was stratified into quintiles: poorest, poorer, middle, richer, and richest [6, 13]. Bangladesh had seven administrative divisions during BDHS 2014: Dhaka, Chittagong, Rajshahi, Khulna, Barishal, Rangpur, and Sylhet [13]. BDHS 2017–18 had one additional division, Mymensingh [6].

### Statistical analysis

First, we described sociodemographic characteristics of the study sample using weighted percentages (%) and unweighted numbers (n) for categorical variables and mean with standard errors for continuous variables. After describing the purpose of using mobile phones for pregnancy-related causes during the last pregnancy, the prevalence (with 95% confidence interval (CI)) of this usage was reported according to background characteristics. To investigate the associated factors of mobile phone usage, we conducted multi-level logistic regression. Similarly, to investigate the association of using mobile phone with at least 4 ANC visits and hospital delivery, we used multilevel logistic regression. Variables that had significance level up to 0.2 during their unadjusted logistic regression analysis with mobile phone use, at least 4 ANC visits, and hospital delivery were adjusted into multivariable analysis [17]. Both unadjusted odds ratio (UOR) and adjusted odds ratio (AOR) with 95% CI were reported. We accounted for the clustering by geographic region (i.e., random intercept) and sample weights of the survey to obtain and report the estimates and used variance inflation factors to assess multicollinearity. Stata 14.0 (College Station, TX, USA) was used to analyze data [18].

## Results

The analysis included a total of 9368 participants, 4465 in the BDHS 2014 and 4903 in the BDHS 2017–18 (Table 1). The average age of the respondents was about 25 years. About 40% of the women were primi during both survey periods. The proportion of women with work status, higher education level, higher educated husbands, 4 or more ANC visits, and hospital delivery was higher in BDHS 2017–18 than BDHS 2014. More than two-thirds of the women were from rural areas in both years. The proportion of people in user group was slightly higher in BDHS 2014 than BDHS 2017–18, 28.5% and 26.6%, respectively.

**Table 1:** Sociodemographic characteristics of the study participants by survey period, % (n) (N = 8876) BDHS 2014 and BDHS 2017–18.

Variables		BDHS 2014	BDHS 2017–18	p-values
		(n = 4465)	(n = 4903)	
Current age of women (in year)	Mean (SE)	24.60 (0.1)	24.89 (0.1)	**<0.001**
	15–24	54.5 (2452)	52.9 (2570)	0.49
	25–34	39.5 (1745)	41.2 (2027)	
	35–49	6.0 (268)	5.9 (306)	
Parity	Primi	40.0 (1816)	38.1 (1864)	0.089
	2 or More	60.0 (2649)	61.9 (3039)	
Currently working	No	76.3 (3488)	62.8 (3066)	**<0.001**
	Yes	23.7 (977)	37.2 (1837)	
Women’s education level	No Education	14.2 (603)	6.2 (303)	**<0.001**
	Primary	28.0 (1228)	27.6 (1361)	
	Secondary	47.7 (2115)	48.9 (2344)	
	College/Above	10.2 (519)	17.2 (895)	
Husband’s education level	No Education	23.9 (1023)	13.7 (677)	**<0.001**
	Primary	30.1 (1348)	33.7 (1647)	
	Secondary	31.6 (1407)	34.1 (1623)	
	College/Above	14.4 (687)	18.5 (956)	
Religion	Muslim	91.7 (4107)	91.9 (4488)	0.93
	Other	8.3 (358)	8.1 (415)	
Household Wealth Status	Poorest	21.7 (936)	20.7 (1060)	0.79
	Poorer	18.9 (849)	20.4 (989)	
	Middle	19.1 (856)	19.0 (878)	
	Richer	20.6 (941)	20.4 (974)	
	Richest	19.6 (883)	19.6 (1002)	
Place of residence	Urban	26.0 (1437)	26.8 (1686)	0.89
	Rural	74.0 (3028)	73.2 (3217)	
Division of residence	Dhaka	35.3 (789)	25.6 (724)	**<0.001**
	Chittagong	21.8 (856)	21.2 (816)	
	Barisal	5.8 (529)	5.7 (521)	
	Khulna	8.0 (527)	9.2 (510)	
	Rajshahi	10.0 (541)	11.6 (515)	
	Rangpur	9.7 (547)	10.6 (547)	
	Sylhet	9.3 (676)	7.6 (680)	
	Mymensingh	0.0 (0)	8.5 (590)	
Ever used mobile phone in pregnancy	No	71.5 (3135)	73.4 (3582)	0.20
	Yes	28.5 (1330)	26.6 (1321)	
Four or more ANC visit	No	68.8 (3039)	52.9 (2537)	**<0.001**
	Yes	31.2 (1426)	47.1 (2366)	
Place of delivery	Home	61.4 (2684)	50.0 (2432)	**<0.001**
	Health facility	38.6 (1781)	50.0 (2471)	

Presented as weighted numbers and unweighted percentages.

Abbreviations: ANC: Antenatal care, BDHS: Bangladesh Demographic and Health Survey, NA: Not available, SE: Standard error.

**Fig 1:**
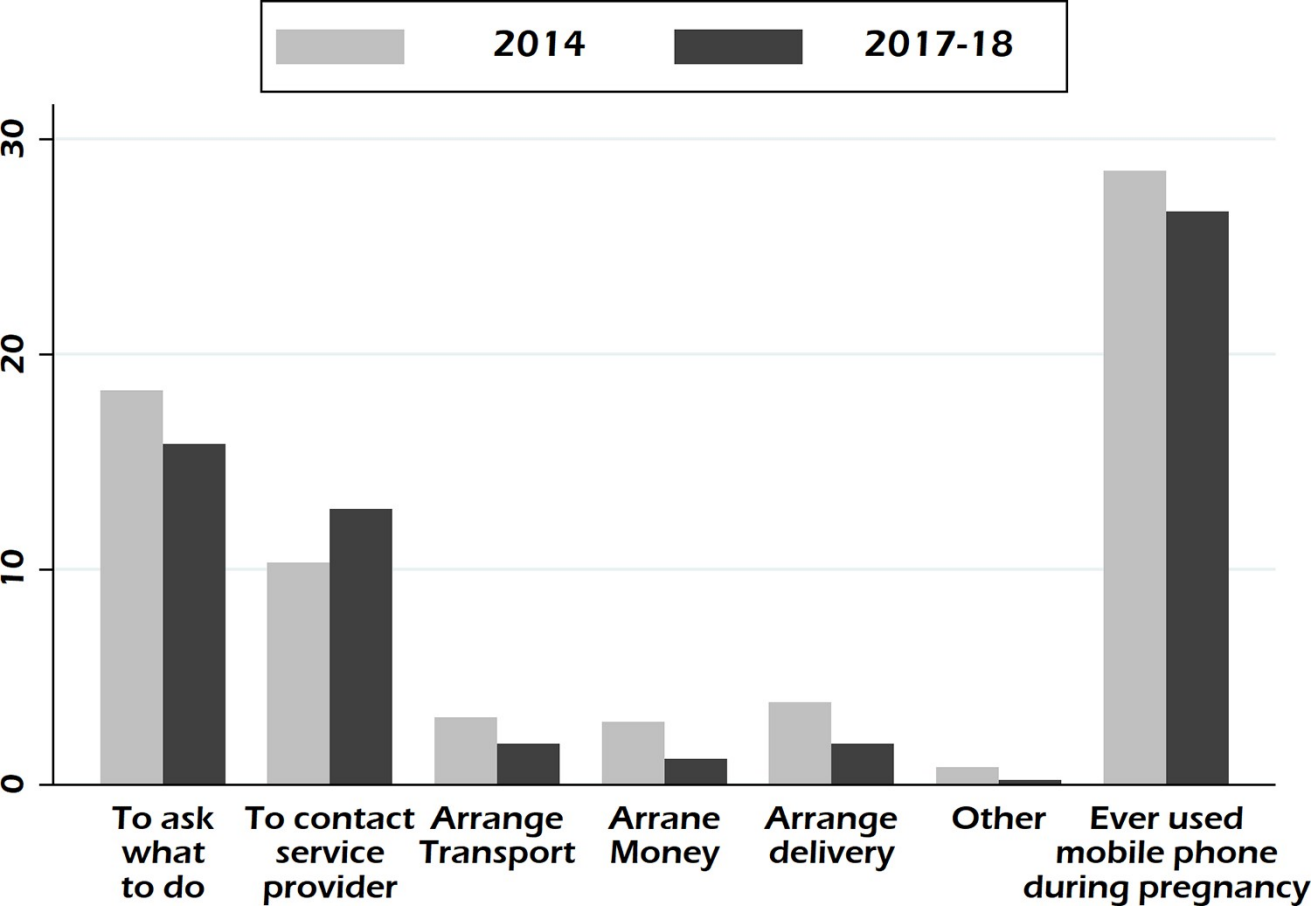
Reasons of using mobile phones for pregnancy-related causes during last pregnancy, Bangladesh Demographic and Health Survey 2014 and 2017–18.

In both surveys, a higher proportion of respondents called “to ask what to do” than all other reasons, 18.3% in BDHS 2014 and 15.8% in BDHS 2017–18, followed by the reason “to contact service provider”, 10.3% in BDHS 2014 and 12.8% in BDHS 2017–18 (Fig 1). A smaller proportion of respondents contacted to arrange money, transport, or place of delivery.

The proportions and trends of ever using mobile phone for pregnancy-related causes according to background characteristics of the respondents are shown in Table 2. Overall, the proportions did not change by age, parity, or husband’s education. The proportion of mobile phone using declined significantly among women with no work, secondary education level, residence in Chittagong and Rajshahi divisions (p<0.05).

**Table 2:** Socio-demographic distribution of mobile phone usage prevalence (95% CI) among respondents for pregnancy by survey period, BDHS 2014 and BDHS 2017–18.

Variables		BDHS 2014	BDHS 2017–18	p-values
Age (in Year)	15–24	28.4(25.7, 31.2)	26.8(24.7, 29.0)	0.38
	25–34	29.8(26.9, 32.8)	26.9(24.5, 29.4)	0.14
	35–49	21.1(15.9, 27.4)	23.5(18.5, 29.4)	0.55
Parity	Primi	33.4(30.1, 36.8)	31.3(28.9, 33.8)	0.33
	2 or More	25.2(23.0, 27.7)	23.8(21.9, 25.8)	0.35
Respondent currently working	No	29.8(27.4, 32.2)	26.5(24.6, 28.5)	**0.039**
	Yes	24.3(21.1, 27.8)	26.8(24.3, 29.5)	0.24
Education Level	No Education	13.1(9.8, 17.2)	14.7(10.6, 20.0)	0.59
	Primary	20.2(17.1, 23.7)	19.1(16.6, 21.9)	0.62
	Secondary	33.9(31.3, 36.6)	27.1(25.0, 29.3)	**<0.001**
	College/Above	47.4(41.7, 53.1)	41.5(37.6, 45.5)	0.096
Husband’s Education Level	No Education	16.9(14.0, 20.2)	15.5(12.5, 19.0)	0.55
	Primary	24.6(21.7, 27.7)	21.8(19.5, 24.3)	0.16
	Secondary	32.4(29.2, 35.8)	28.3(25.8, 30.9)	0.054
	College/Above	47.3(41.7, 52.9)	40.6(36.9, 44.4)	0.053
Religion	Muslim	28.4(26.3, 30.6)	26.6(24.9, 28.3)	0.21
	Other	29.7(23.6, 36.7)	27.3(22.0, 33.2)	0.59
Household Wealth Status	Poorest	18.1(15.0, 21.7)	19.0(16.0, 22.4)	0.085
	Poorer	22.6(19.4, 26.2)	21.9(19.0, 25.0)	0.64
	Middle	28.9(23.8, 34.6)	27.4(23.9, 31.1)	0.73
	Richer	31.0(27.2, 34.9)	28.8(25.6, 32.1)	0.39
	Richest	42.5(37.6, 47.6)	36.7(32.8, 40.8)	0.075
Place of Residence	Urban	33.8(29.9, 38.0)	29.5(26.4, 32.8)	0.10
	Rural	26.6(24.3, 29.0)	25.6(23.7, 27.6)	0.51
Division of Residence	Dhaka	26.1(22.7, 29.7)	30.3(26.3, 34.7)	0.13
	Chittagong	34.6(29.1, 40.4)	22.7(19.5, 26.3)	**0.001**
	Barisal	30.9(25.2, 37.4)	32.8(25.8, 40.6)	0.71
	Khulna	29.5(25.1, 34.4)	31.2(26.8, 35.9)	0.61
	Rajshahi	27.4(23.1, 32.3)	20.3(16.3, 25.1)	**0.03**
	Rangpur	29.7(25.9, 33.8)	32.8(27.8, 38.3)	0.35
	Sylhet	20.7(15.3, 27.4)	18.9(15.7, 22.6)	0.62
	Mymensingh	NA	24.0(19.7, 28.9)	NA

Abbreviations: ANC: Antenatal care, BDHS: Bangladesh Demographic and Health Survey, CI: Confidence interval, NA: Not available.

Table 3 shows the results of unadjusted and adjusted multilevel logistic regression analyses to identify factors associated with mobile phone use for pregnancy-related causes in BDHS 2014 and BDHS 2017–18. The AOR was higher among women with primi (i.e., 1^st^ pregnancy) than 2^nd^ or more pregnancy history in BDHS 2014 (AOR: 1.5, 95% CI: 1.3–1.7) and BDHS 2017–18 (AOR: 1.2, 95% CI: 1.1–1.4). Women with higher education levels, higher educated husbands, or higher wealth status had higher odds of using mobile phones than those with lower education or wealth. Although respondents residing in the Chittagong division had higher odds in BDHS 2014 (AOR: 1.6, 95% CI: 1.3–1.9) than Dhaka, the odds were lower in BDHS 2017–18 (AOR: 0.7, 95% CI: 0.5–0.9). Barisal had greater odds both in BDHS 2014 (AOR: 1.4, 95% CI: 1.1–1.8) and BDHS 2017–18 (AOR: 1.4, 95% CI: 1.1–1.8) than Dhaka. Only in BDHS 2014, the odds were lower among residents in Rajshahi (AOR: 0.7, 95% CI: 0.5–0.9) and Sylhet (AOR: 0.7, 95% CI: 0.5–0.8) divisions while these were higher among respondents from Rangpur (AOR: 1.4, 95% CI: 1.1–1.8) division.

**Table 3:** Factors associated with mobile phone use for pregnancy-related causes by survey period, BDHS 2014 and BDHS 2017–18.

Variables		BDHS 2014		BDHS 2017–18	
		COR (95% CI)	AOR (95% CI)^1^	COR (95% CI)	AOR (95% CI)^1^
Age (in Year)	15–24	1.0 (Ref.)	1.0 (Ref.)	1.0 (Ref.)	
	25–34	1.0 (0.9,1.2)	1.3*** (1.1,1.6)	1.0 (0.9,1.2)	
	35–49	0.8 (0.6,1.1)	1.3 (1.0,1.9)	0.9 (0.7,1.2)	
Parity	Primi	1.6*** (1.4,1.8)	1.5*** (1.3,1.8)	1.5*** (1.3,1.7)	1.2** (1.1,1.4)
	2 or More	1.0 (Ref.)	1.0 (Ref.)	1.0 (Ref.)	1.0 (Ref.)
Work Status	No	1.0 (Ref.)	1.0 (Ref.)	1.0 (Ref.)	
	Yes	0.8** (0.7,1.0)	0.9 (0.8,1.1)	1.1 (0.9,1.2)	
Education Level	No Education	1.0 (Ref.)	1.0 (Ref.)	1.0 (Ref.)	1.0 (Ref.)
	Primary	1.6*** (1.2,2.1)	1.4* (1.1,1.9)	1.4 (1.0,1.9)	1.2 (0.8,1.7)
	Secondary	3.4*** (2.6,4.4)	2.4*** (1.8,3.2)	2.0*** (1.4,2.8)	1.5* (1.0,2.1)
	College/Above	6.1*** (4.5,8.2)	2.8*** (1.9,4.0)	3.9*** (2.7,5.4)	2.1*** (1.4,3.1)
Husband’s Education Level	No Education	1.0 (Ref.)	1.0 (Ref.)	1.0 (Ref.)	1.0 (Ref.)
	Primary	1.5*** (1.2,1.8)	1.1 (0.9,1.4)	1.5*** (1.2,1.9)	1.3* (1.0,1.7)
	Secondary	2.4*** (2.0,2.9)	1.3 (1.0,1.6)	2.0*** (1.6,2.6)	1.5** (1.1,1.9)
	College/Above	4.1*** (3.3,5.1)	1.5** (1.1,2.0)	3.3*** (2.6,4.2)	1.7*** (1.2,2.3)
Religion	Muslim	1.0 (Ref.)	1.0 (Ref.)	1.0 (Ref.)	
	Other	1.2 (0.9,1.5)	1.1 (0.9,1.4)	1.0 (0.8,1.3)	
Household Wealth Status	Poorest	1.0 (Ref.)	1.0 (Ref.)	1.0 (Ref.)	1.0 (Ref.)
	Poorer	1.4* (1.1,1.7)	1.1 (0.9,1.4)	1.1 (0.9,1.4)	1.0 (0.8,1.2)
	Middle	1.9*** (1.5,2.4)	1.3* (1.0,1.6)	1.6*** (1.3,2.0)	1.3 (1.0,1.6)
	Richer	2.3*** (1.8,2.8)	1.4** (1.1,1.8)	1.7*** (1.3,2.0)	1.2 (1.0,1.5)
	Richest	4.0*** (3.2,5.0)	1.9*** (1.5,2.6)	2.4*** (2.0,3.0)	1.5** (1.1,1.9)
Place of Residence	Urban	1.0 (Ref.)	1.0 (Ref.)	1.0 (Ref.)	1.0 (Ref.)
	Rural	0.7*** (0.6,0.8)	1.0 (0.8,1.2)	0.8** (0.7,0.9)	1.0 (0.9,1.2)
Division of Residence	Dhaka	1.0 (Ref.)	1.0 (Ref.)	1.0 (Ref.)	1.0 (Ref.)
	Chittagong	1.5*** (1.2,1.8)	1.6*** (1.3,1.9)	0.7*** (0.5,0.8)	0.7** (0.5,0.9)
	Barisal	1.2 (0.9,1.5)	1.4** (1.1,1.8)	1.2 (0.9,1.5)	1.4* (1.1,1.8)
	Khulna	1.2 (1.0,1.5)	1.2 (1.0,1.6)	1.2 (0.9,1.5)	1.2 (0.9,1.6)
	Rajshahi	1.1 (0.8,1.3)	1.2 (0.9,1.5)	0.6*** (0.5,0.8)	0.7** (0.5,0.9)
	Rangpur	1.0 (0.8,1.3)	1.2 (0.9,1.5)	1.2 (1.0,1.5)	1.4* (1.1,1.8)
	Sylhet	0.7** (0.6,0.9)	0.9 (0.7,1.2)	0.5*** (0.4,0.7)	0.7** (0.5,0.8)
	Mymensingh	NA	NA	0.7* (0.6,0.9)	0.9 (0.7,1.1)

Abbreviations: AOR: Adjusted odds ratio, ANC: Antenatal care, BDHS: Bangladesh Demographic and Health Survey, CI: Confidence interval, COR: Crude odds ratio, NA: Not available

*: p<0.05

**: p<0.01

***:p<0.001.

Adjusted for all variables in the columns, some variables from unadjusted odds ratio in 2017–18 were not adjusted as the p-values were above 0.20.

In BDHS 2014, 43.3% (95% CI: 38.8–47.8) of women received at least 4 ANC visits among user group while this proportion was 26.4% (95% CI: 23.8–29.1) among non-user group (Table 4). In 2017–18, among both groups, the proportion increased, 59.1% (95% CI: 55.7–62.4) and 42.8% (95% CI: 40.4–45.2) among the user and non-user groups, respectively. The adjusted odds of at least 4 ANC were higher in BDHS 2014 (AOR: 1.6, 95% CI: 1.4–1.9) and BDHS 2017–18 (AOR: 1.4, 95% CI: 1.3–1.7) among users than non-users. Additionally, the proportion and adjusted odds of receiving hospital delivery were higher among users than non-users.

**Table 4:** Association of mobile phone using for pregnancy-related causes with at least 4 ANC visits and facility delivery.

Variables		BDHS 2014			BDHS 2017–18		
		Proportion among ever-user	COR (95% CI)	AOR (95% CI)^1^	Proportion among ever-user	COR (95% CI)	AOR (95% CI)^2^
Four or More ANC Visits							
Ever use Mobile Phone	No	26.4 (23.8, 29.1)	1.0 (Ref.)	1.0 (Ref.)	42.8 (40.4, 45.2)	1.0 (Ref.)	1.0 (Ref.)
	Yes	43.3 (38.8, 47.8)	2.3***(2.0,2.6)	1.6***(1.4,1.9)	59.1 (55.7, 62.4)	1.8***(1.6,2.1)	1.4***(1.3,1.7)
Facility Delivery							
Ever use Mobile Phone	No	31.2 (28.0, 34.6)	1.0 (Ref.)	1.0 (Ref.)	45.1 (42.6, 47.5)	1.0 (Ref.)	1.0 (Ref.)
	Yes	57.0 (52.7, 61.2)	3.0***(2.6,3.5)	2.0***(1.7,2.4)	63.8 (60.1, 67.3)	2.1***(1.8,2.4)	1.5***(1.3,1.8)

Abbreviations: AOR: Adjusted odds ratio, ANC: Antenatal care, BDHS: Bangladesh Demographic and Health Survey, CI: Confidence interval, COR: Crude odds ratio, NA: Not available

*: p<0.05

**: p<0.01

***: p<0.001.

Adjusted for current age, parity, education level, husband’s education, religion, household wealth status, and place and division of residence.

Adjusted for current age, parity, four or more antenatal care visits, education level, husband’s education, religion, household wealth status, and place and division of residence.

## Discussion

This study investigated the patterns and factors associated with using mobile phones for pregnancy-related causes and how this use is associated with at least 4 ANC visits and hospital delivery after analyzing two nationally representative survey datasets from Bangladesh, BDHS 2014 and 2017–18. We found that women with primi gravida, higher socioeconomic status (i.e., higher education level, husbands with higher education level, and higher wealth quintiles), and residence in some administrative divisions had higher odds of using mobile phones for pregnancy-related causes. Using mobile phones was also associated with higher likelihoods of at least 4 ANC visits and hospital delivery. Our study provided a potential opportunity to generate evidence for incorporating the dynamic tool of mobile phone technology to improve the access, health care seeking approach, and maternal health care utilization practices in Bangladesh.

Overall, only a little higher than one-fourth of the total respondents used mobile phones for pregnancy-related causes in both survey years. With increasing ownership and subscription, we expected a simultaneous increase in that, however, the change was in opposite direction. Higher maternal health services (i.e., ANC and hospital delivery) utilization among women with the history of mobile phone use for pregnancy-related causes have important implications for the country. Studies from other LMICs had similar findings, therefore, access to and use of mobile phone for pregnancy-related causes may increase maternal health care utilization in other countries as well [8, 19–21]. The Government of Bangladesh has initiated a toll-free national emergency service hotline (i.e., 999) since 2016, Goal 3 of the SDG is also one of the related goals of the initiative [22]. As information related to maternal health care are available through that helpline, scaling up of these already available services could improve the overall utilization.

The socioeconomic differences in mobile phone using are also observed in utilization of maternal health care utilization [23, 24]. Historically, studies that investigated the association of socioeconomic status with maternal health services utilization found a consistently lower utilization among women with a poor socioeconomic background [25]. As these population groups are also at a higher risk of maternal and neonatal deaths, mobile phone use could serve as an intervention to improve maternal health care utilization among these population groups [26]. Mobile phone use could also contribute to the reduction of socioeconomic disparities in maternal health care utilization and outcomes. It is important to reach them with appropriate programs and policies. Similar socioeconomic differences in health care utilization, health outcomes, and mobile phone use have been reported from other countries [8, 19–21]. Therefore, uptake of mobile phone use can increase in mobile phone use in those countries as well. Many national and international nongovernmental organizations are currently working in partnership with the government to increase uptake of these services; some of them are using mHealth services as well [27, 28]. A positive association of using mobile for pregnancy-related causes with health services suggests that expanding these mHealth services are likely to result in an uptake of the maternal health care utilization.

We also observed differences in using mobile phone according to administrative divisions similar to prior studies [15, 29, 30]. Similar to socioeconomic differences between regions, the divisional differences in health care utilization and health outcomes have been observed [6, 23, 25]. Regional differences have been observed in many other countries as well. Previous studies also reported differences in mobile phone ownership and access by regions [16, 21]. Seeking care depends on multiple factors and interactions between factors. In addition to socioeconomic status, to reach out to a service provider, maternal autonomy plays a significant role. Women with higher autonomy can seek care for themselves without solely depending on their spouse or other family members [31, 32]. These women are more likely to own mobile phones as well [16]. Women with higher socioeconomic status also have higher autonomy [6]. Interventions aiming to increase access, ownership, or use of mobile phones should consider these interactions of factors. A large proportion of women are engaged with non-governmental organizations in Bangladesh, interventions through these NGOs can improve the mobile phone utilization for pregnancy-related causes.

The ‘three delays’ model is commonly mentioned to describe delays in accessing maternal health care. These delays are: (1) “delay in decision to seek care”; (2) “delay in reaching care”; and (3) “delay in receiving adequate health care” [33]. Expanding mobile phone use for pregnancy-related causes therefore can reduce the valuable time wasted in first and second delays. In other words, using mobile phones to seek information about health services, contact the service providers, manage money, arrange transport to go health centers, and set up place of delivery can substantially reduce the time to seek and reach care. The third delay in accessing care should also be considered. BDHS 2017–18 reported that quality ANC was received by only 18% of the women; socioeconomic disparities were observed in receiving quality ANC too [6]. Only 13% women went to public sector health facilities. Services offered by public health facilities are available with a minimal fee in Bangladesh [6]. We primarily examined quantity of services, assessing quality of services and the reasons for not utilizing that were beyond the scope of our study; future studies should assess the quality of services provided by all public health centers using some common benchmarks, especially in regions with lower maternal health service utilization. It is also important to examine the reasons for not utilizing these freely available services. Effective programs and policies should be developed to assess, expand, and improve these services.

This study has several notable strengths. We analyzed two nationally representative datasets with large sample sizes and high response rates to investigate the trends and associations. To minimize recall bias, we only included the history of last pregnancy. The survey used standardized and validated questionnaires that increased authenticity of our findings [6, 13]. However, limitations of the present study also merit discussion. The datasets were cross-sectional, the studied associated factors and confounders (e.g., wealth quintile) were the data during the time of survey, not necessarily during the pregnancy time [6, 13], therefore, the observed association may not be causal due to lack of a temporal relationship [34]. Data on several important variables (e.g., delivery complications) were not available and that may impact the observed association.

## Conclusion

This study demonstrated that using mobile phones for pregnancy-related causes can improve maternal health services utilization in Bangladesh. Considering the high MMR and NMR along with a low maternal health service utilization, effective programs, policies, and interventions should be implemented to increase use of mobile phones for maternal health care utilization, particularly among women belonging to lower socioeconomic groups.

## Supporting information

S1 Fig Flow diagram of the data extraction process. (TIFF)

S2 Fig Conceptual framework for the relationship of mobile phone use with antenatal care visits and hospital delivery. (TIFF)
